# Fungus Fighters: Wood Ants (*Formica polyctena*) and Their Associated Microbes Inhibit Plant Pathogenic Fungi

**DOI:** 10.1007/s00248-024-02464-2

**Published:** 2024-11-21

**Authors:** Ida Cecilie Jensen, Andreas Schramm, Joachim Offenberg

**Affiliations:** 1 Terrestrial Ecology, Department of Ecoscience, Aarhus University, Aarhus, Denmark; 2 Section for Microbiology, Department of Biology, Aarhus University, Aarhus, Denmark

**Keywords:** Biological control, Pant diseases, Antimicrobial microorganisms, *Formica polyctena*, Pathogen suppression, Biopesticides

## Abstract

Plant diseases cost the global economy billions of US dollars every year. The problem has mainly been addressed by using chemical pesticides, but recently, the use of ants has shown promising effects against plant pathogens. However, the mechanisms accounting for these effects have not yet been determined. One possible explanation is antimicrobial microorganisms associated with ants. Through controlled laboratory experiments, we investigated the inhibitory effects of wood ants (*Formica polyctena*) and their associated microorganisms against economically important plant pathogenic fungi. All live ants, extracts from crushed ants, and extracts from washed ants significantly inhibited the apple brown rot (*Monilinia fructigena*) while yielding the growth of other microbes. Furthermore, all investigated wood ants transferred microorganisms to their surroundings within 10 s when walking across a surface. We isolated the most dominant microorganisms deposited by walking ants and from washed ant extracts (i.e., strains likely found on the surface of ants), resulting in four bacterial cultures and one yeast. Two of these isolates, strain I3 (most closely related to *Pseudomonas sichuanensis* and *P. entomophila*) and strain I1b (most closely related to *Bacillus mycoides*), showed inhibitory effects against apple brown rot and apple scab (*Venturia inaequalis*), while strain I3 also inhibited gray mold (*Botrytis cinerea*) and *Fusarium* head blight (*Fusarium graminearum*). These results suggest that wood ants have potential as biological control agents against commercially relevant plant pathogens, and that their inhibitory effect might be at least partially caused by antibiotic compounds produced by their associated microorganisms.

**Supplementary Information:**

The online version contains supplementary material available at 10.1007/s00248-024-02464-2.

## Introduction

The field of plant pathogen control holds innovative potential. Every year, 20–40% of the global crop production is lost due to plant diseases and pest insects [1]. The most widely applied solution for protection is chemical pesticides, which are effective and easily applied but have several negative effects, including pesticide resistance [2], human health issues [3], and adverse effects on biodiversity [4–7]. In the search for sustainable alternatives to chemical pesticides, the use of insects, in particular ants, has attracted increasing attention.

Ants have long been considered effective at controlling a wide variety of pest insects [8]. However, recent studies have shown that the presence of ants may also lower incidences of plant pathogens. A recent literature review found 13 ant species that were able to control 14 fungal, oomycete, and bacterial plant pathogens on 12 different plant species, including agricultural crops [9]. As an example, the presence of ants in European fruit orchards lowered incidences of pear scab (*Venturia pyrina*) [10], apple scab (*Venturia inaequalis*) [11], and apple brown rot (*Monilinia fructigena*) [12]. Similar results have been found on mango trees, coffee shrubs, and oak seedlings, where the presence of weaver ants (*Oecophylla smaragdina*), *Crematogaster* spp., and *Crematogaster scutellaris* ants reduced incidences of mango scab (*Elsinoë mangiferae*) [13], coffee leaf rust [14], and leaf fungal attacks [15], respectively. Understanding the mechanisms underlying these effects could improve the use of ants as a potent tool in crop protection.

Three main hypotheses have been proposed to explain how ants can reduce plant pathogens: i) ants forage and eat fungal spores, thereby actively removing them from the plants [16, 17]; ii) ants deter insects that either vector or facilitate pathogens, reducing the pathogen incidences on associated plants [18, 19]; and lastly, iii) chemicals produced by the ants or their associated microorganisms have antimicrobial effects [9, 20]. The latter mechanism holds substantial potential to explain some of the observed effects.

The production of antimicrobial chemicals is an important tool that enables ants to prevent diseases. Ants live in dense communities consisting of closely related individuals, which increases the risk of contracting and transmitting diseases [21]. Thus, ants have evolved an arsenal of chemical and behavioral defense mechanisms to combat pathogens. For example, some ants have metapleural glands, which produce antibiotic compounds [17, 22–24]. Interestingly, these ant-produced compounds often have broad-spectrum effects, and, in some cases, they also inhibit plant pathogens. A recent review summarized a total of 22 ant-produced chemicals that were able to inhibit 20 different plant pathogens [20]. These compounds could potentially be transferred or actively secreted to the ants’ surroundings, protecting not only the ants from pathogens but also their environment.

Antimicrobial compounds are not only produced by the ants themselves but also by associated microorganisms residing on the ants. In the same review, Offenberg et al. [20] reported 30 ant symbionts that inhibited 10 different plant pathogens. As with the ant-produced compounds, these antimicrobial compounds could also be transferred passively to the ants’ environment, especially if the microorganisms are located on the ants’ surface. For example, González-Teuber et al. [25] isolated seven antimicrobial bacterial strains from the legs of two ant species (*Pseudomyrmex ferrugineus* and *Pseudomyrmex gracilis*)*.* Thus, antimicrobial microorganisms from the ants’ legs could protect the plants that ants roam from pathogens.

The antimicrobial effects of ants and their associated microbes may add a new service to the use of ants in biological control, as ants might serve as potent tools against plant pathogens while simultaneously combatting pest insects [26].

Based on field experiments showing a correlation between wood ant (*Formica polyctena*) presence and reduced apple disease incidences [11, 12], we aim to investigate the cause of this inhibitory effect of wood ants*.* Through controlled laboratory experiments, we test whether wood ants can inhibit plant pathogens and if potential effects could be explained by their associated microorganisms. To separate effects from ants and their surface-associated microorganisms, we assess the inhibitory effects of i) live ants, ii) extracts from crushed ants, and iii) extracts from washed ants. Inhibition is tested against apple brown rot (*M. fructigena*)*,* which is one of the pathogens affected by ants in the field. Additionally, we examine the transfer of microorganisms from ants to their environment and test the inhibitory effects of the most prevalent microorganisms isolated from the ants’ surface, i.e., from washed ant extracts, and the microorganisms deposited from the feet of walking ants. These isolates are then tested against *M. fructigena* and three other economically important plant pathogens: Apple scab (*V. inaequalis*), gray mold (*Botrytis cinerea*), and Fusarium head blight (*Fusarium graminearum*). In doing so, we aim to elucidate one of the potential mechanisms underlying the antimicrobial effect of wood ants, which could provide new insights for improved implementation of ants as crop protection in agriculture.

## Materials and Methods

### Cultivation of Ants and Fungi

*M. fructigena* (DSM 2677)*, V. inaequalis* (DSM 1002)*, B. cinerea* (DSM 877)*,* and *F. graminearum* (DSM 1095) were purchased from the German collection of microorganisms and cell cultures (DSMZ). *M. fructigena* was inoculated on Petri dishes containing either tomato agar (TA) (200 mL of organic tomato passata (strained tomatoes), 3.0 g of calcium carbonate (CaCO_3_), 15 g of agar, and 1000 mL Milli-Q water) or malt peptone agar (MPA) (30 g of malt extract (Sigma-Adrich), 3 g of tryptic soy broth (Scharlau), 15 g of agar, and 1000 mL Milli-Q water), while *V. inaequalis* was inoculated on MPA, and *B. cinerea* and *F. graminearum* were inoculated on potato dextrose agar (PDA) (24 g potato dextrose broth (Scharlau), 15 g agar, and 1000 mL Milli-Q water). All media were autoclaved at 121 °C for 15 min. Inoculated agar plates were incubated at 23 °C in the dark. When hyphal growth was visible, the process was repeated. Ultimately, agar plates with visible hyphal growth were stored at 5 °C until use.

*F. polyctena* ants (hereafter wood ants) [27] were sampled from three laboratory colonies kept in 65 L mortar tubs at a greenhouse (Aarhus University, Department of Ecoscience, Vejlsøvej 25, 8600 Silkeborg, Denmark). Each colony was kept in its own mortar tub containing pine needles, nest material, and sugar and water feeders. The colonies were regularly fed mealworms for protein. Each colony was originally sampled from a wild colony found in Silkeborg, Denmark, and had been kept in the lab for 4–6 months. Three wood ant colonies were used for the three experiments, so a different colony was used for each experiment.

### Inhibitory Tests with Ants

To test the inhibitory effect of live ants, three wood ants were added to each of 23 two-compartment TA plates (Fig. S1). Ants only had access to one compartment in each dish, while the other was an ant-free control. Ants were present on the agar for 24 h, after which all compartments were inoculated with *M. fructigena.* The *M. fructigena* inoculant was prepared by adding 5 mL of sterilized Milli-Q water to agar plates with visible *M. fructigena* hyphal growth. Hyphae were gently dislodged using an inoculation loop. Subsequently, 25 µL of the solution was added to each compartment of the dishes. The solution was evenly distributed with a Drigalski spatula. Agar plates were incubated at 23 °C in darkness. Inhibition was assessed after four days when hyphal growth was visible in the control and defined as no *M. fructigena* growth. Photos of all replicates from this and the following experiments are provided in the Supplementary information (Fig. S2-5).

We also tested the inhibitory effect of whole, crushed ants against *M. fructigena*. An extract was prepared by adding five live ants to an Eppendorf tube containing 500 µL of sterilized Milli-Q water and two glass beads. The Eppendorf tube was shaken vigorously by hand for 2–3 min until the ants had disintegrated. The inhibitory effect of the extract was tested against *M. fructigena* cultures prepared as above. Then, 50 µL of the pathogen solution was evenly distributed onto each of the 20 TA plates. Each agar plate was divided into four sectors, each with a paper disc; two discs were treated with ant extract, while the other two served as controls. Paper discs (diameter, 15 mm, Whatman® qualitative filter paper discs) were autoclaved for 15 min at 121 °C, placed in the middle of each sector with sterilized forceps [28], and either 20 µL of ant extract or sterilized Milli-Q water was added. Subsequently, agar plates were incubated at 23 °C in darkness. Inhibition was assessed after four days and defined as a clear inhibition zone around the filter paper discs (Fig. S6). Growth of bacteria and fungi from the ant extracts on the filter discs was inspected visually and by phase contrast microscopy. Cases where fungal growth did not reach the filter paper without the presence of an inhibition zone were considered inconclusive (Fig. S3-4).

Lastly, we tested the inhibitory effect of washed ant extract and microorganisms. This experiment was conducted as the one above. However, instead of crushed ants, inoculants were prepared by adding five live ants to an Eppendorf tube containing 500 µL of sterilized Milli-Q water. The solution was shaken carefully without glass beads for 30 s without breaking the ant bodies. Then, 20 µL of this solution was pipetted onto sterilized paper discs together with controls as above. Incubation and inhibitory assessments were done as described above.

### Ant-Associated Microorganisms

To test whether ants transfer microorganisms to their environment, a single ant was added to each of 10 agar plates for 10 s. The agar plates were prepared with two types of media: six contained TA, and four contained MPA. After 10 s, the ants were removed, and the agar plates were incubated as above until microbial growth was visible. This experiment used ants from the same colony as the 24-h live ant activity experiment.

The two most abundant microorganisms from these tests and the three dominant ones from the filter discs with washed ant extract were then transferred onto MPA and TA plates. As all isolates grew equally well on the two media, subsequent transfers were made on MPA plates due to their light color, making growth easier to assess. The plates were incubated as above for four days; then, single colonies were picked and repeatedly re-streaked to purity.

The five isolates (four bacteria and one yeast; Fig. S7) were tentatively identified by colony-PCR amplification and Sanger sequencing of their 16S rRNA gene (for bacteria; primers 8F/1492R) or the partial 28S rRNA gene (for the yeast; primers ITS1/LR3) as described in detail previously [29]. The almost full-length 16S rRNA gene sequences and the fungal partial 28S rRNA gene sequence have been deposited at NCBI under accession numbers PP545489-PP545492 (bacteria) and PP545504 (yeast).

The inhibitory effect of the five isolates was tested against *M. fructigena* and *V. inaequalis* on MPA and against *B. cinerea* and *F. graminearum* on PDA plates. However, as one of the bacterial strains could not be grown on PDA, the effect of this isolate was tested on MPA for all four pathogens. For each pathogen (except *V. inaequalis,* see below), the microbial isolates were inoculated in four spots (one in each quarter of the plate) on four agar plates each and contrasted with four control plates containing only the pathogens. Inoculation solutions with *M. fructigena, B. cinerea*, and *F. graminearum* were prepared and incubated as above, and inhibition was defined as a pathogen*-*free zone around the isolates. Plates with *V. inaequalis* were prepared by cutting two small pieces (approximately 0.5 cm in diameter) of agar with *V. inaequalis* growth and putting them on top of an MPA plate, one in each half. Each isolate was then inoculated in a line between the two pieces on three plates each. These plates were contrasted with three control plates containing only *V. inaequalis*. Plates were incubated as above, and inhibition was defined as those cases where *V. inaequalis* grew only in the opposite direction of the isolate (away from the midline) or showed no growth at all. Photos of all inhibitory tests are provided in the Supplementary information (Fig. S8-11).

### Statistical Analyses

Bayesian statistical methods were used to test the inhibitory effects in all analyses. We tested the inhibitory effect of crushed ant extract, washed ant extract, and live ants against *M. fructigena*. Models used inhibition of *M. fructigena* growth as a binary response and treatment (ants or control) as a categorical explanatory variable. A Bernoulli distribution was used as the response was binary, and the explanatory variables only had two levels. All models used a normal prior over the finite interval of (0, 100) for all fixed effects. Models were tested both with inconclusive data included in the models as “no inhibition” (i.e., the value 0) and as “NA” (i.e., data was excluded from the analyses).

Hamiltonian Markov Chain Monte Carlos (MCMC) was performed for each model, using four chains with 20,000 iterations, of which 10,000 were warmups. The summary statistics of the resulting fits were extracted, and credible intervals were used to assess the effect of the variables. All analyses were performed using R-studio version 2021.09.0 (R version 4.3.1), with the packages brms (version 2.16.1) [30] and RcmdrMisc (version 2.7–1), and the R script is provided in the Supplementary information. Effects were considered significant when both the lower and upper 95% credible intervals were either above or below zero, as the parameter value zero was defined as no effect [31]. Thus, intervals below zero indicate significant inhibition. As the resulting 95% credible intervals were significant and very similar in range both when including and excluding inconclusive data, we chose to present the analyses where inconclusive data is included as “no inhibition” (For results of the excluding analyses, Table S1).

## Results

### Inhibition from Ants

All three experiments testing the inhibitory effect of live ants, crushed ant extract, and washed ant extract showed significant inhibition of *M. fructigena*. These results are summarized in Table 1.

**Table 1:** Inhibition data of the three experiments: live ants, crushed ant extract, and washed ant extract. “A” refers to ant treatments, while “C” refers to controls without ants**.** Numbers before the slash indicate the number of either agar plates or filter papers showing inhibition or overgrowth, while numbers after are the total sample size. Inconclusive papers/plates are presented in the table. Credible intervals for the models of the three laboratory experiments, where L-CI_95%_ denotes the lower 95% credible interval and U-CI_95%_ denotes the upper 95% credible interval. Significance is defined as both lower- and upper 95% credible intervals above or below zero, here, written in bold

	Inhibition results				Statistical analyses		
	Inhibition		Overgrown				
Experiment	A	C	A	C	Variables	L-CI 95%	U-CI 95%
Live ant activity	14/21	0/21	0/21	14/21	Ants vs. control	** − 25.84**	** − 3.26**
Crushed ant extract	32/40	0/40	2/40	30/40	Ants vs. control	** − 27.23**	** − 4.69**
Washed ant extract	34/40	0/40	4/40	36/40	Ants vs. control	** − 27.22**	** − 5.00**

**Fig. 1:**
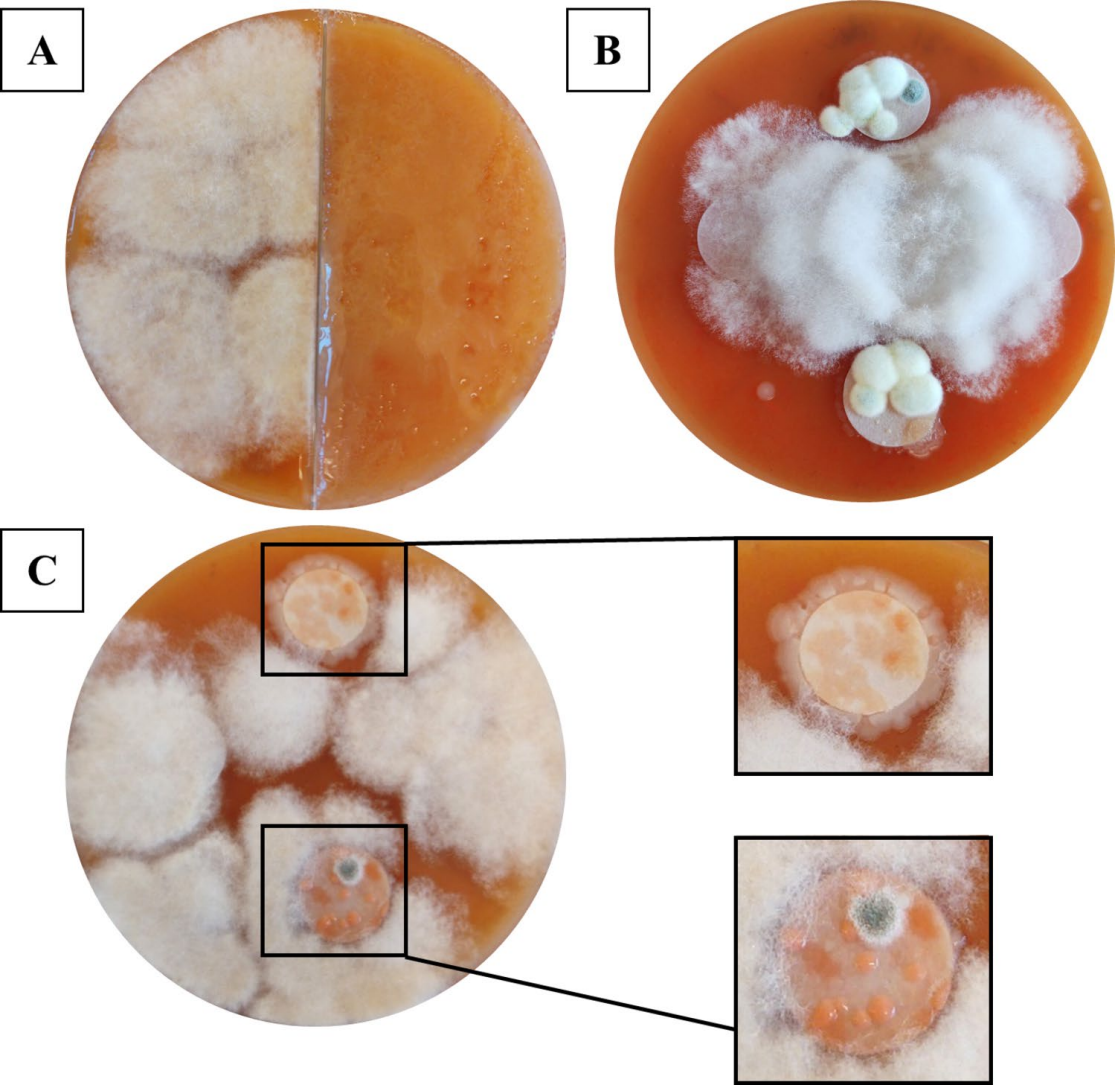
Agar plates showing the results of **A** the live ant activity, **B** crushed ant extract, and **C** washed ant extract. For **A**, the left side of the agar plate shows the ant-free control compartment, while the right side shows the ant compartment, where three ants have been roaming for 24 h. For **B**, the two vertical filter papers were treated with crushed ant extract; they show growth of fungal and bacterial colonies from the extract. The two horizontal filter papers were treated with sterilized Milli-Q water and were overgrown with *M. fructigena* (the white, fluffy fungus growing circularly across the agar plates). For **C**, vertical filter papers were treated with washed ant extract, while overgrown horizontal filter papers were Milli-Q controls. The right side of **C** shows a zoomed-in picture of the inhibition zones around the filter papers and of the microbial growth on the paper. All replicates for each experiment can be found in the Supplementary information

The 24 h of live ant activity on agar significantly inhibited the growth of *M. fructigena* (Table 1, *CI*_*95%*_ = [− 25.84, − 3.26]) (Fig. 1A). A total of 12 out of 21 ant compartments had complete absence of fungal growth, while two of the remaining showed inhibition via a reduced fungal growth compared to controls. Most of the compartments with inhibited pathogen growth showed a lawn or biofilm of microbial growth (Fig. 1A). The last seven compartments were inconclusive, as there was no growth of *M. fructigena* in the control compartments. Except for these seven, all other control compartments showed *M. fructigena* growth. Two agar plates were removed from the analysis as an ant escaped into the control compartment during preparation (Fig. S2).

The crushed ant extract significantly inhibited the growth of *M. fructigena* (Table 1, *CI*_*95%*_ = [− 27.23, − 4.69]) (Fig. 1B). Inhibition was observed on 32 of the 40 filter papers with ant extract. Two papers were overgrown by *M. fructigena*, while six were inconclusive. In contrast, none of the 40 control filter papers showed inhibition; 30 were overgrown (Fig. S3), whereas 10 were inconclusive due to the lack of *M. fructigena* growth on the agar (Fig. S3). All ant extract papers had clear fungal and/or bacterial growth, often consisting of a few morphologically similar microbes. Thirty-nine out of 40 ant extract papers showed growth of a fungus, which by visual inspection was tentatively identified as *Penicillium* sp., as it resembled other *Penicillium* species in coloration and conidiophore structure (Fig. S12) [32].

Lastly, the washed ant extract also significantly inhibited the growth of *M. fructigena* (Table 1, *CI*_*95%*_ = [− 27.22, − 5.00]) (Fig. 1C). Inhibition was visible on 34 of 40 ant extract papers. Four papers were overgrown and two inconclusive. None of the control papers showed inhibition; 36 were overgrown and four were inconclusive (Fig. S4). All ant extract papers showed apparent microbial growth, dominated by three morphotypes: an orange-pinkish yeast (on 35 of 40 papers; Fig. S13A), a yellow-pinkish bacterium (on 34 of 40 papers; Fig. S13B), and a white bacterial colony (on 15 of 40 papers; Fig. S13C). One representative of each morphotype was isolated, resulting in isolates I1a, I4, and I1b.

#### Ant-Associated Microorganisms and Their Antimicrobial Effect

Based on their partial 28S rRNA and near-complete 16S rRNA gene sequences, respectively (Table 2), the three dominating isolates from the washed ant extract were identified as most closely related to the yeast *Rhodotorula alborubescens* (isolate I1a), and the two bacteria *Asaia spathodeae* (I4) and *Bacillus mycoides* (I1b).

**Table 2:** Identity, source, and frequency of the isolated microorganisms and their inhibitory effect against the four plant pathogens

Isolate	I1a	I1b	I2	I3	I4
Closest relative(s)	*Rhodotorula alborubescens*	*Bacillus mycoides*	*Scandinavium goeteborgense* and *Pantoea endophytica*	*Pseudomonas sichuanensis* and *P. entomophila*	*Asaia spathodeae*
% sequence identity/bp^a^	28S rRNA: 99.6/590	16S rRNA: 100/1513	16S rRNA: 98.23/1542, 98.15/1422	16S rRNA: 99.93/1525, 99.13/1526	16S rRNA: 100/1414
Source & frequency^b^	Washed ant filters: 35/40	Washed ant filters: 15/40	Transfer test plates: 5/10	Transfer test plates: 5/10	Washed ant filters: 34/40
Inhibition against plant pathogens^c^					
*M. fructigena*	-	+	-	+	-
*V. inaequalis*	-	+	-	+	-
*B. cinerea*	-	-	-	+	-
*F. graminearum*	-	-	-	+	-

^a^Sequence identity in % for the compared gene (28S rRNA or 16S rRNA) and number of base pairs (bp)

^b^Number of filters or plates on which the respective morphotype was found out of the total number of samples

^c^—indicates no inhibition, + indicates inhibition of the respective plant pathogen

**Fig. 2:**
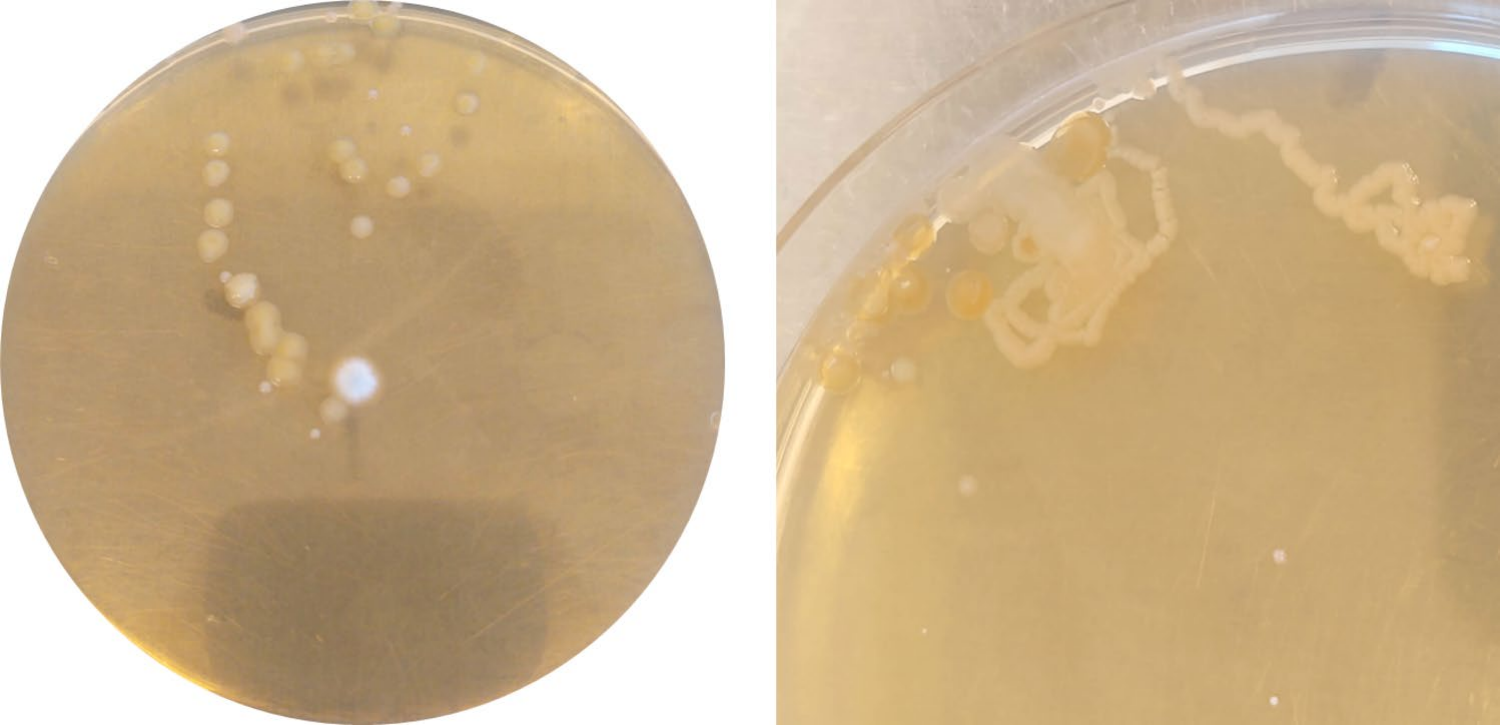
Agar plates from the transfer test showing examples of microbial growth after 10 s of ant activity

In the transfer test, 10 s of ant activity left a visible trail of microbial growth on all 10 agar plates (Fig. 2, Fig. S5). Two bacterial morphotypes, a light-yellow and a dark-brown one, were both found on 5 of the 10 agar plates and sometimes co-occurred (Fig. S14). A representative of each morphotype was isolated. Based on its 16S rRNA gene sequence, the first isolate (I2) was most similar to *Scandinavium goeteborgense* and *Pantoea endophytica*, while the second (I3) was closely related to *Pseudomonas sichuanensis* and *Pseudomonas entomophila* (Table 2).

Two of the five isolates, i.e., *Pseudomonas* I3 and *Bacillus* I1b, were able to inhibit some of the plant pathogens (Table 2). *Pseudomonas* I3 inhibited all four plant pathogens, while *Bacillus* I1b inhibited *M. fructigena* and *V. inaequalis*. The remaining three isolates did not inhibit any of the plant pathogens (Table 2). *Pseudomonas* I3 showed an especially strong inhibitory effect as it more or less eliminated all *M. fructigena* growth on the agar plates (Fig. S8).

## Discussion

Field studies have shown that incidences of *M. fructigena* on apple trees were reduced when wood ants were present in the orchard [12]. However, the mechanisms accounting for this effect have not yet been established. Our study supports this observation, as ants significantly inhibited the in vitro growth of *M. fructigena.* This suggests a potential causal link between ant presence and the observed reduction in the field.

There are multiple ways in which ants can cause an antimicrobial effect. Firstly, ants excrete gland contents, including formic acid from the poison gland and trail pheromones deposited during trail laying, which are both known to possess antibiotic properties [33–36]. Secondly, ants are known to be associated with antimicrobial microorganisms residing on their bodies [20]. In our 24-h live ant experiment, microbial growth in the form of colonies and biofilms was visible on the plates, suggesting that the ants transferred microorganisms during the 24 h. These microorganisms could originate from within the ants (deposited through regurgitation and feces) or from the exterior of the ants (deposited through walking). Additionally, ants may also deposit gland excretions, e.g., trail pheromones and formic acid, while roaming the agar. Consequently, the antimicrobial effect observed in this experiment might be caused both by gland excretions and antimicrobial microorganisms. However, based on the microbial growth on the plates, it seems unlikely that gland excretions were the only source of antimicrobial compounds. Associated microorganisms could also play an important role in the observed antimicrobial effect, but to apply this effect in practice, we need to understand where the microorganisms are located and how they are transferred to the environment.

Diverse microbial growth and inhibition of *M. fructigena* was visible in both the crushed ant and washed ant extracts. Crushed ant extracts have previously been found to inhibit plant pathogens as Zhang [37] and Alagappan et al. [38] showed that crushed weaver ant (*Oecophylla smaragdina*) extracts significantly inhibited the growth of *M. fructigena* and *Staphylococcus aureus*, respectively. However, the effect of gland contents and associated microorganisms cannot be separated when ants are crushed. By carefully washing the ants, gland excretions become less abundant in the extract. Despite this reduction of gland excretions, we found that the washed ant extract still inhibited *M. fructigena* growth. These results suggest that the antimicrobial effect may originate, at least partly, from microorganisms on the exterior of the ants.

The presence of antimicrobial microorganisms on the ants is further supported by our isolation of two highly antimicrobial bacteria from the washed ant extract and the transfer test. Both bacteria were most likely situated on the exterior of the ants, as *Pseudomonas* sp. I3 was isolated from ant footprints, while *B. mycoides* strain I1b was carefully washed of the exterior of the ants, reducing the likelihood of them being internal microbes. This indicates that the ants’ antimicrobial properties may easily be transferred to the surroundings, including to host plants or the plants that ants roam. Furthermore, it is encouraging that the two isolates are closely related to species that have already shown potential against plant pathogens. The *Pseudomonas* sp. I3 is closely related to *P. entomophila*, which can control citrus canker (*Xanthomonas canker*) [39], while *B. mycoides* is effective against the four plant pathogens, *Rhizoctonia solani*, *Fusarium solani*, *Sclerotium rolfsii*, and *B. cinerea* [40, 41]. These findings suggest that antimicrobial microorganisms are present on the exterior of wood ants and highlight their potential in combatting plant pathogens.

Finding antimicrobial microorganisms on wood ant legs is coherent with the findings of González-Teuber et al. [25] who isolated 15 bacterial strains from the legs of two tropical ant species (*Pseudomyrmex ferrugineus* and *Pseudomyrmex gracilis*) of which seven were able to inhibit three microorganisms, including two plant pathogens (*Pseudomonas syringae, Pseudomonas.* sp., and *Escherichia coli*). Together with our findings, this shows i) that ants can carry antimicrobial bacteria on their legs, ii) that these can be effective against plant pathogens, and iii) that they can be deposited where ants walk. This mechanism is not restricted to tropical ant species and may contribute to the negative correlations between ant presence and plant disease incidence observed in in situ studies [9–11, 15].

To improve the implementation of wood ants as crop protection, further studies should investigate whether wood ants, as well as other ant species, are generally associated with antimicrobial microorganisms or if this is restricted to specific geographical regions. This is important, as it might limit the geographical sources where effective ant populations can be obtained. Likewise, further studies should investigate the feasibility of culturing these antimicrobial microorganisms and directly applying them as biopesticides on plants. However, using live ants has the advantage of controlling both plant pathogens and pest insects, making them a potent multitool in crop protection. Conversely, using ants can be troubling as many ant species tend hemipterans, e.g., aphids, which can be pests of crops, but this disservice can be managed by feeding the ants with sugars [10, 11, 42, 43] or by using biocontrol agents targeted towards the aphids [44]. Despite this disservice, the role of ants in agroecosystems tends to be more beneficial than harmful [45].

The results of this study demonstrate the inhibitory effect of wood ants on plant pathogenic fungi. This effect is likely to be at least partly caused by antimicrobial microorganisms associated with the ants, some of which are effective against plant pathogens and passively transferred to the surroundings as microbial trails when the ants walk.

The results of this study are the first demonstration of a potential mechanism underlying the antifungal effect of ants observed in field studies and highlight the great potential of using ants as biological control agents in agriculture.

## Supplementary Information

Below is the link to the electronic supplementary material.

Supplementary file1 (PDF 3584 KB)

## Data Availability

The almost full-length 16S rRNA gene sequences and the fungal partial 28S rRNA gene sequence have been deposited at NCBI under accession numbers PP545489-PP545492 (bacteria) and PP545504 (yeast).
