# Supplementary material for: Fungus Fighters: Wood Ants (Formica polyctena) and Their Associated Microbes Inhibit Plant Pathogenic Fungi
Source: Microb Ecol. 2024 Nov 21;87:146. doi: 10.1007/s00248-024-02464-2 (PMC11582330; doi:10.1007/s00248-024-02464-2)
Supplement: Supplementary file 1 [file 248_2024_2464_MOESM1_ESM.pdf]

Supplementary information for: Fungus Fighters: Wood Ants (*Formica polyctena)* and Their Associated Microbes Inhibit Plant-Pathogenic Fungi

Ida Cecilie Jensen[1,2], Andreas Schramm[2], and Joachim Offenberg[1]

[1]Terrestrial Ecology, Department of Ecoscience, Aarhus University, Aarhus, Denmark.
[2]Section for Microbiology, Department of Biology, Aarhus University, Aarhus, Denmark.

Journal of Microbial Ecology

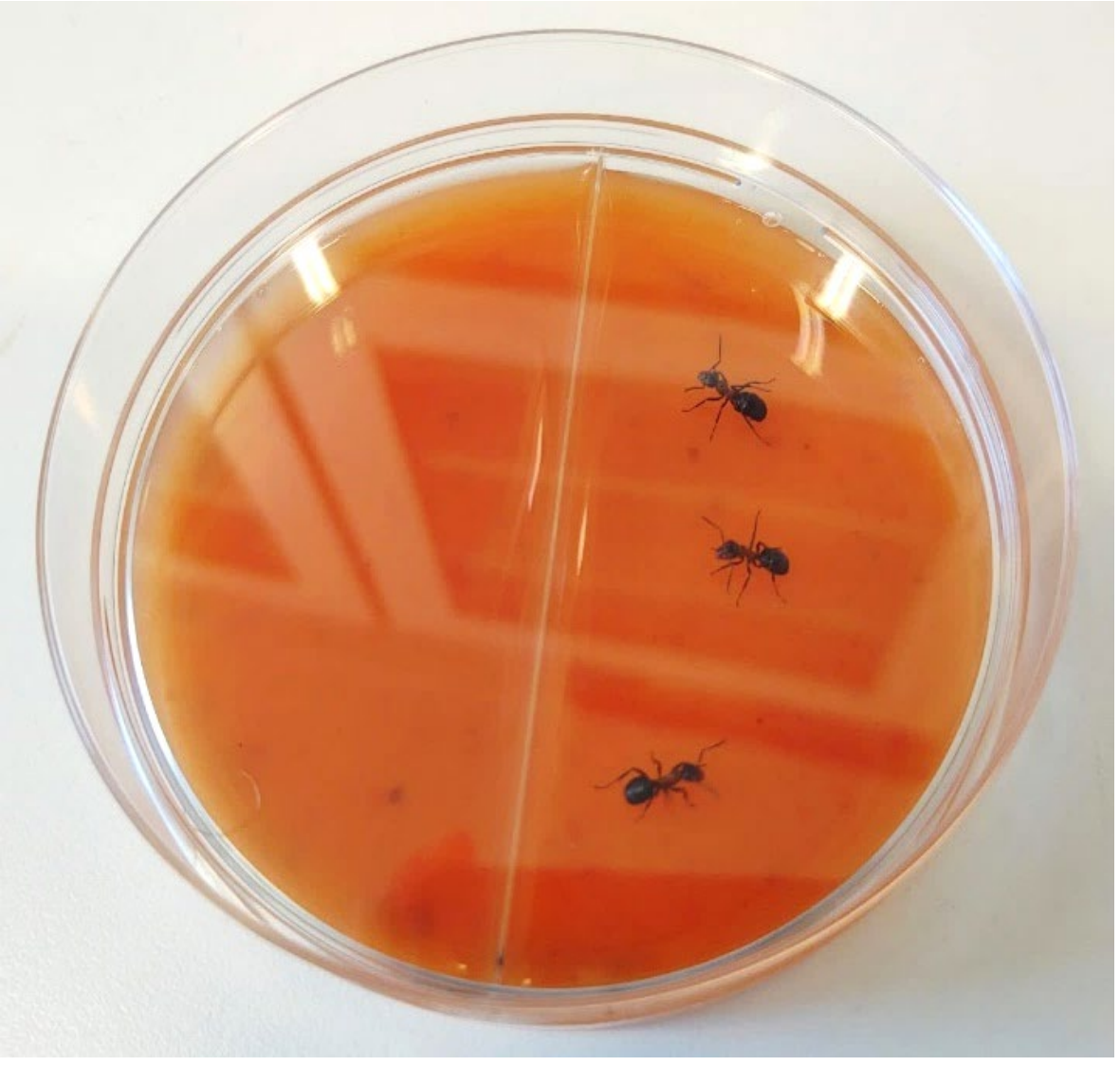

Figure S1. Two compartment Petri dish used to test the prophylactic effect of live wood ants.

Table S1. Inhibition data of the three experiments, crushed ants, ant inoculants, and 24 hours of ant activity, when inconclusive data were excluded from the analyses. Numbers before the slash refer to the ant treatments, while numbers after refer to controls without ants**.** Credible intervals for the models of the three laboratory experiments, where L-CI$_{95\%}$ denotes the lower 95% credible interval, while U-CI$_{95\%}$ denotes the upper 95% credible interval. Significance is defined as both lower- and upper 95% credible intervals above or below zero, here, written in bold.

	Inhibition test results on ant/control plates				Statistical analyses		
**Experiment**	**Inhibition**	**Overgrown**	**Inconclusive**	**Total**	**Variables**	**L-CI 95%**	**U-CI 95%**
24-hour ant activity	14*/0	0/14	7/7	21/21	Ants vs. Control	**-228.20**	**-13.54**
Crushed ant extract	32/0	2/30	6/10	40/40	Ants vs. Control	**-28.19**	**-5.99**
Washed ant extract	34/0	4/36	2/4	40/40	Ants vs. Control	**-27.48**	**-5.39**

*Including 12 with complete inhibition and two with reduced growth of *M. fructigena*.

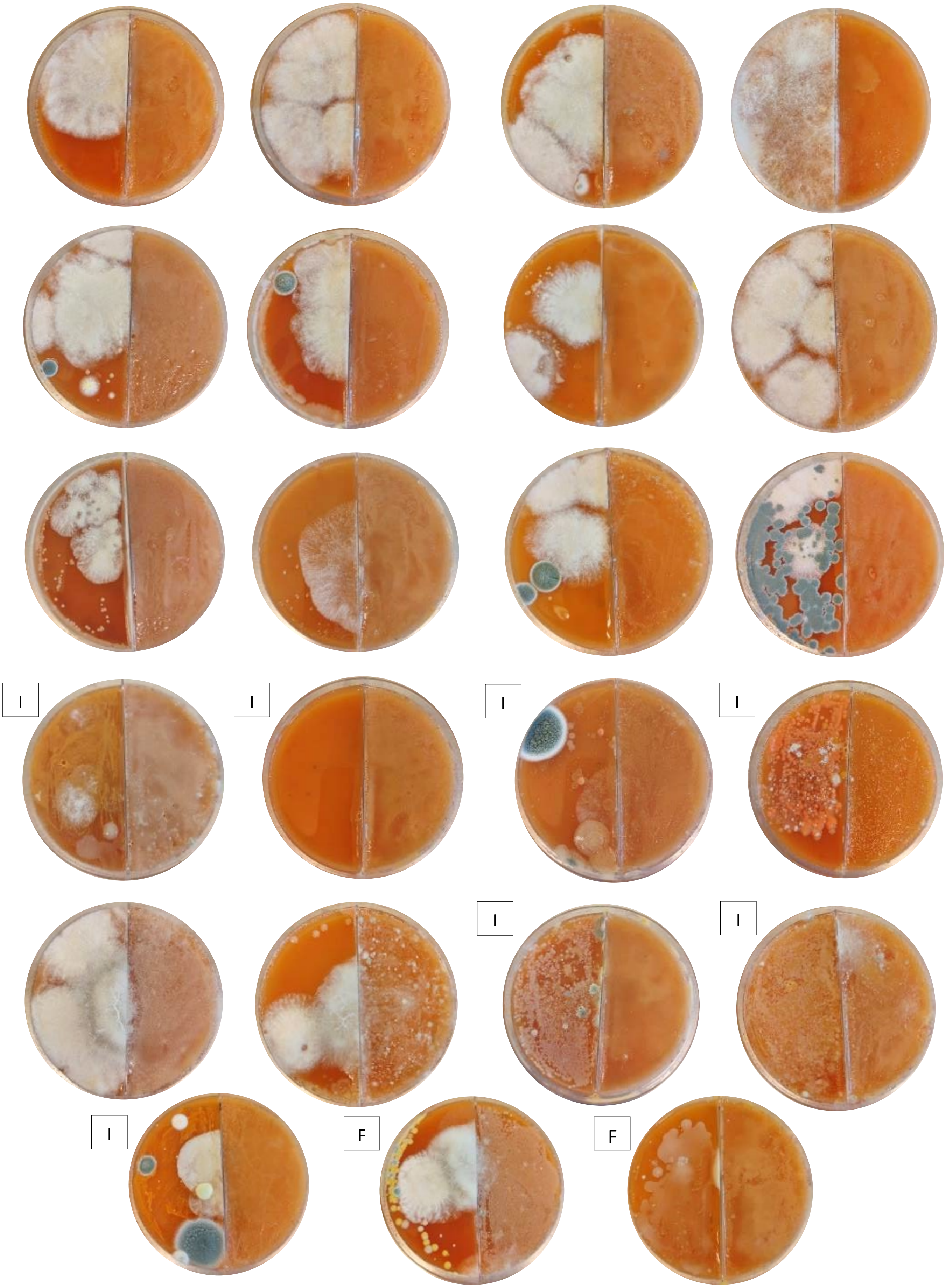

Figure S2. All replicates used for the live ant experiment. Left side of the agar plates show the control compartments without ants, while the right side show the ant compartments where wood ants were roaming for 24 hours. The white, fluffy fungus radiating circularly across the agar plates is apple brown rot *Monilinia fructigena*. Boxes with "I" indicate inconclusive agar plates, while "F" indicates the agar plates that were left out due to ants escaping into the control compartment.

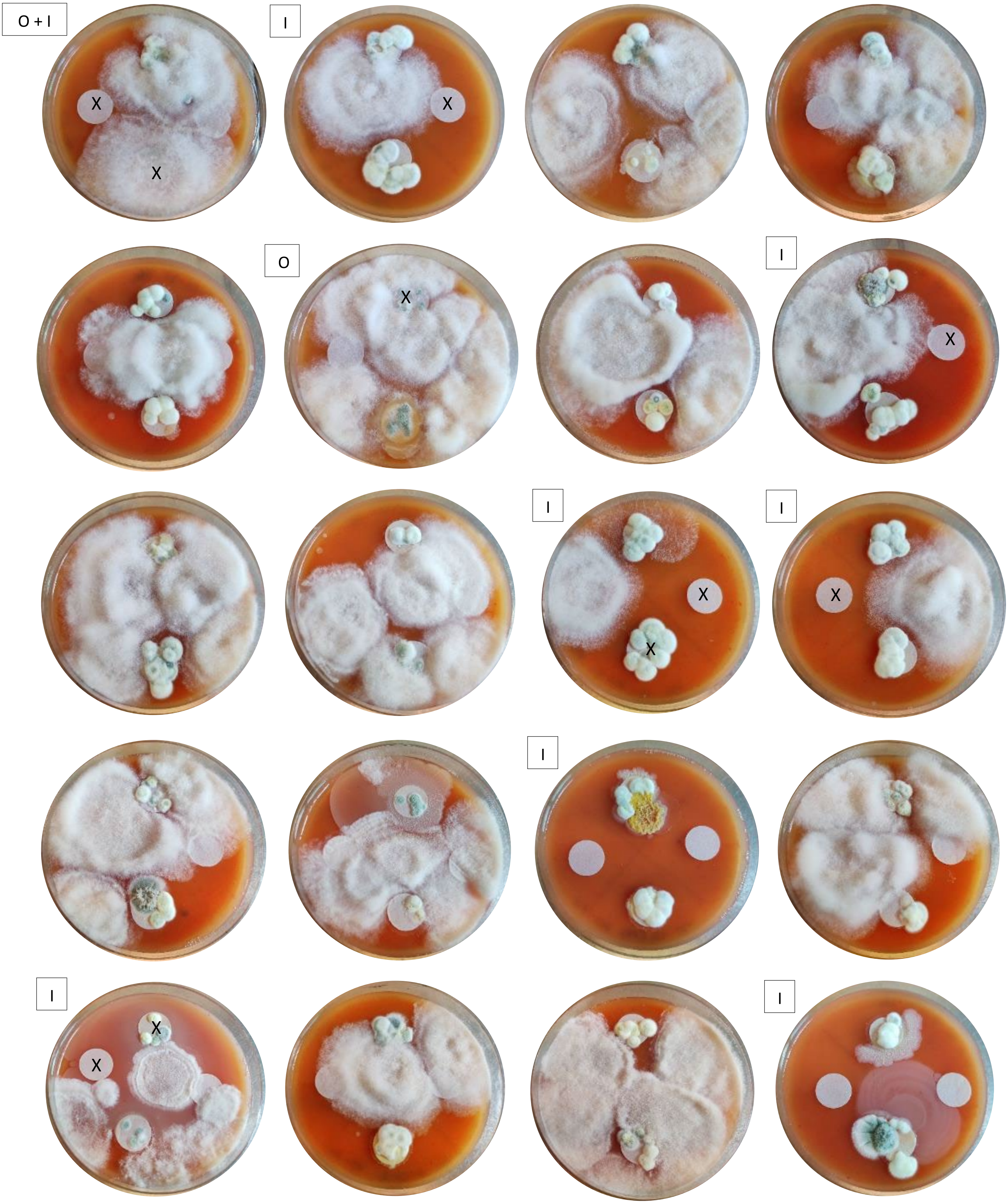

Figure S3. All replicates tested with the crushed ant extract. The two vertical filter papers of each agar plate were treated with crushed ant extract, while the two horizontal filter papers were treated with sterilized Milli-Q water. The white, fluffy fungus growing in large, circular patterns across the agar is *M. fructigena*. Boxes with “I” indicate inconclusive filter papers, while “O” indicate overgrown ant filter papers. “X” indicates which filter papers the “I” or “O” refer to. If no “X” is present, the “I” or “O” refers to all filter papers within the Petri dish.

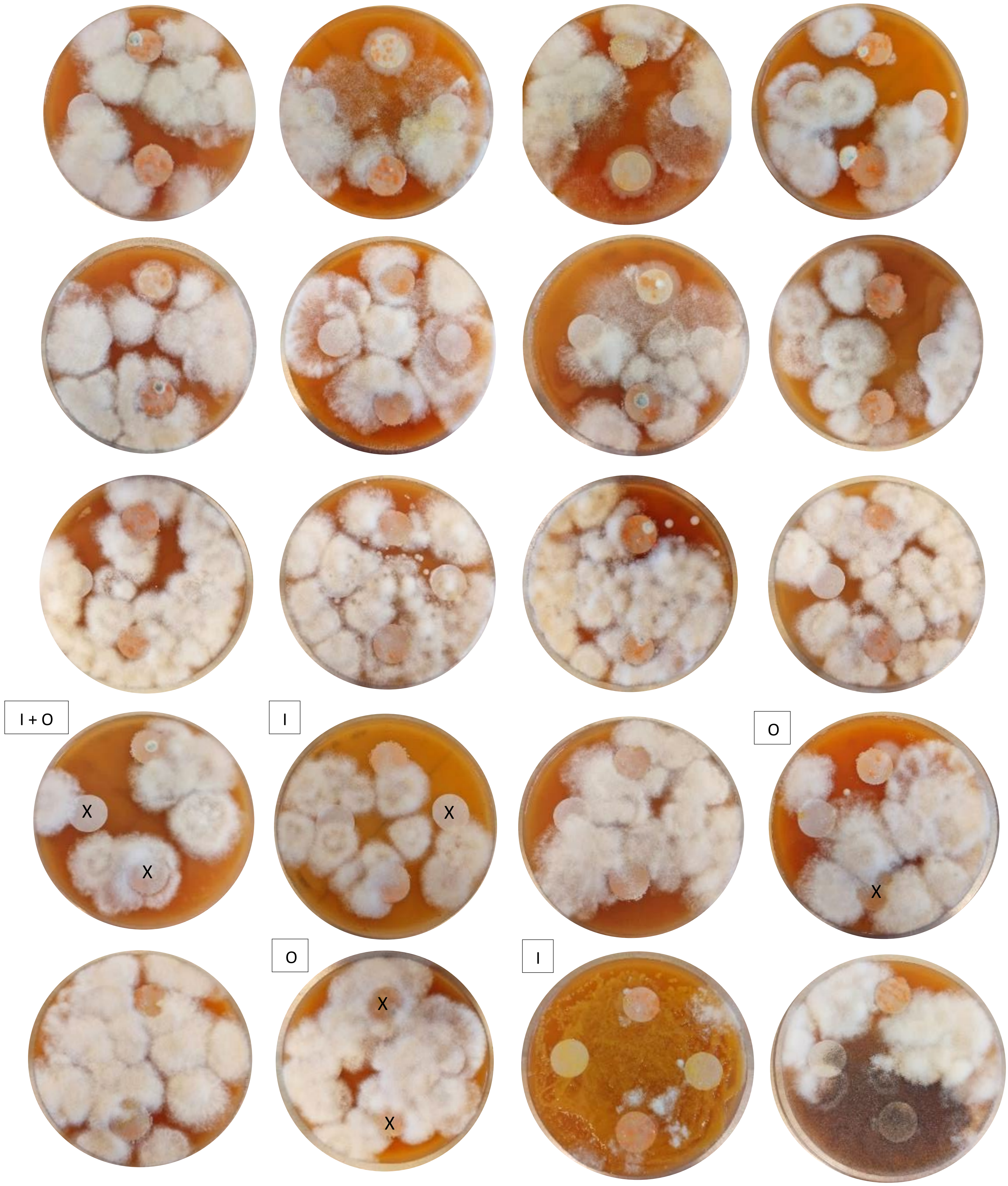

Figure S4. All replicates tested with the washed ant extracts. The two vertical filter papers of each agar plate were treated with the washed ant extract, while the two horizontal filter papers were treated with sterilized Milli-Q water. The white, fluffy fungus growing in large, circular patterns across the agar is *M. fructigena*. Boxes with "I" indicate inconclusive filter papers, while "O" indicate overgrown ant filter papers. "X" indicates which filter papers the "I" or "O" refer to. If no "X" is present, the "I" or "O" refers to all filter papers within the Petri dish.

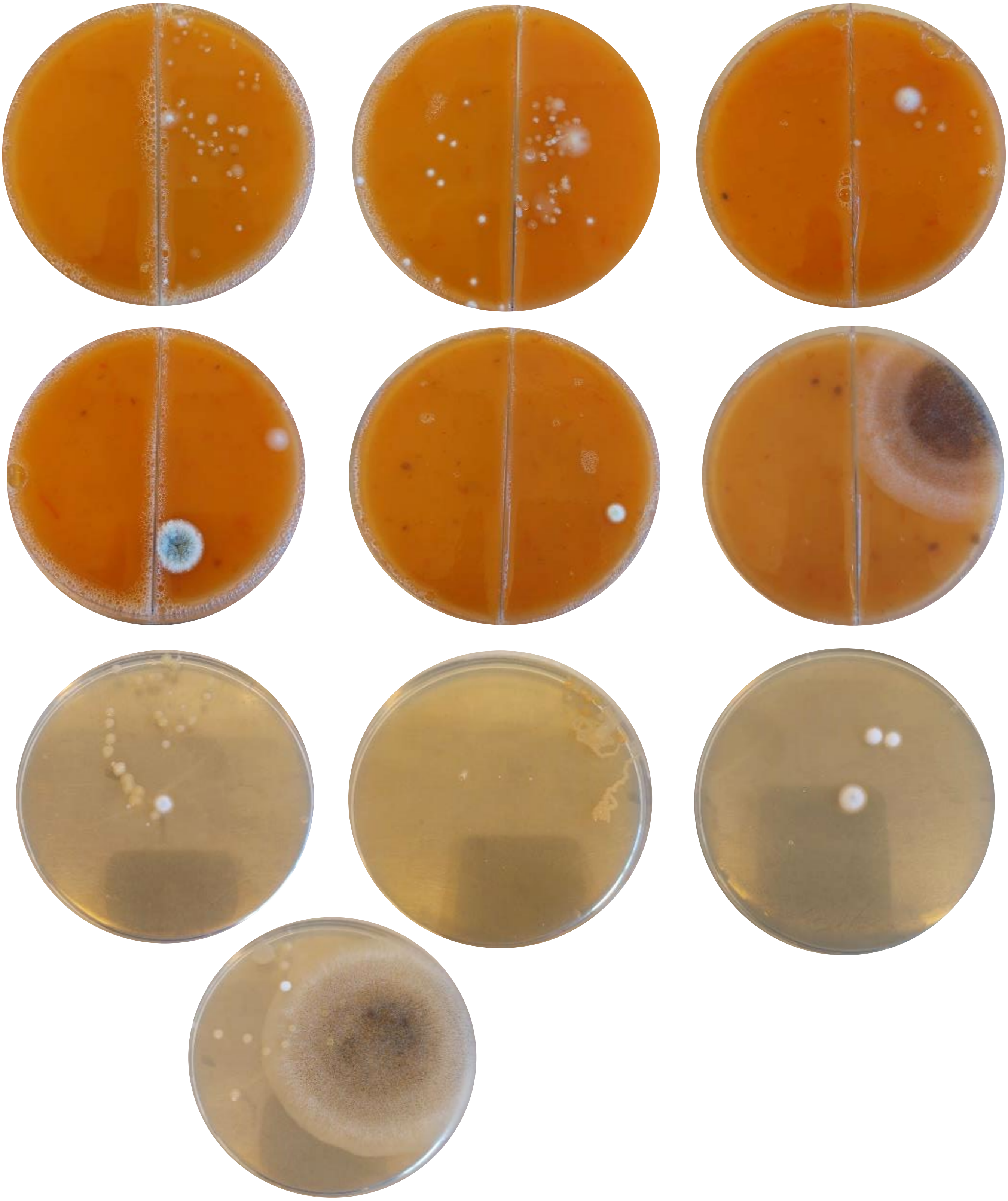

Figure S5. All replicates used for the transfer test. Ants were given 10 seconds to roam the agar, and for the two-compartment Petri dishes, ants were allowed to climb the separating wall.

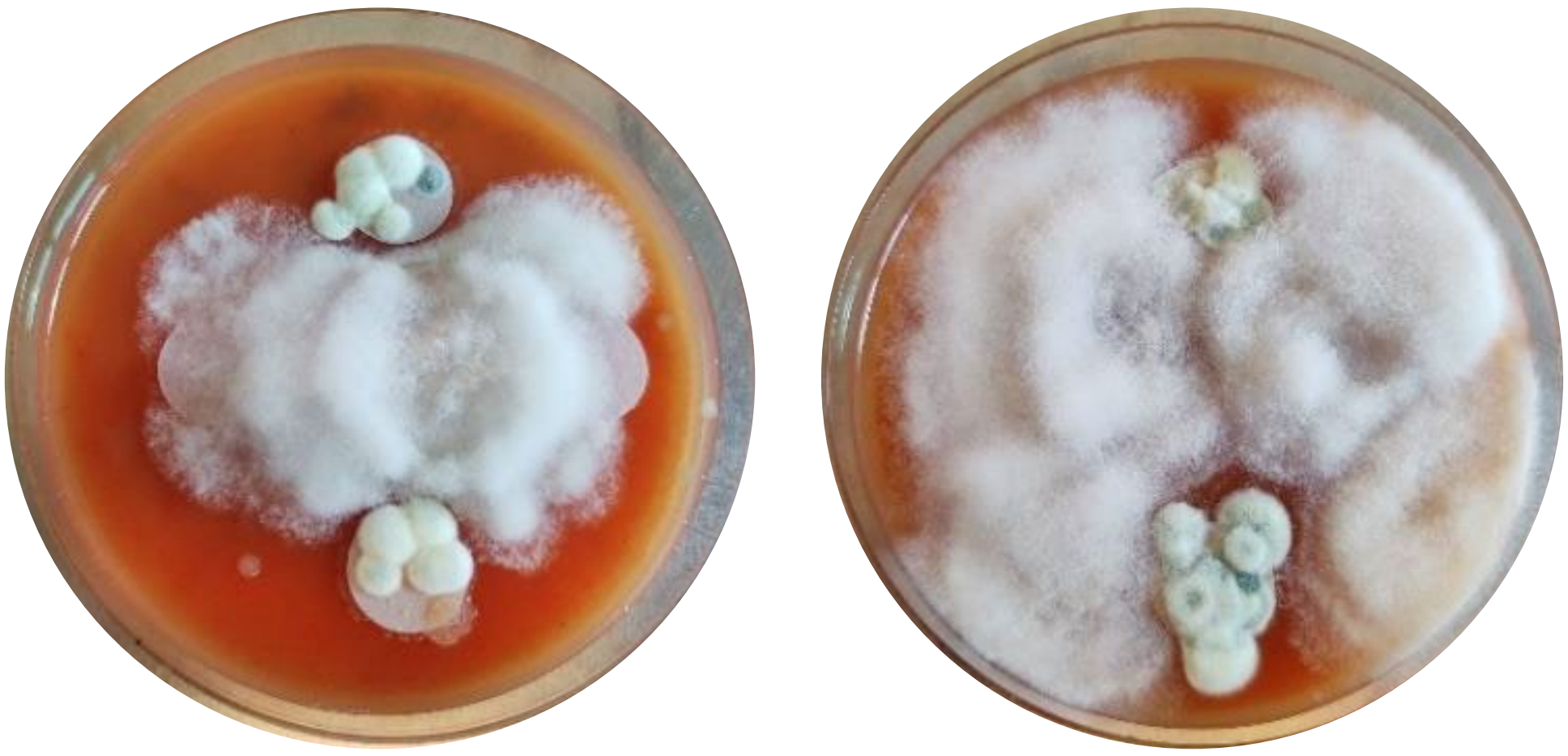

Figure S6. Visual examples of the range of inhibition. The two vertical filter papers of each Petri dish were treated with crushed ant extract, while the two horizontal filter papers were treated with sterilized Milli-Q water. The white, fluffy fungus growing circularly across the agar is *M. fructigena*. The left-side agar plate shows clear inhibition of *M. fructigena* for both vertical wood ant filter paper discs, while control filter papers are completely overgrown. The right-side agar plate shows a similar clear inhibition on the lower ant filter paper, while the upper filter paper is more overgrown. However, as the *M. fructigena* is only taking up the "air space" above the filter paper and not actually growing onto it, this filter paper is also showing inhibition.

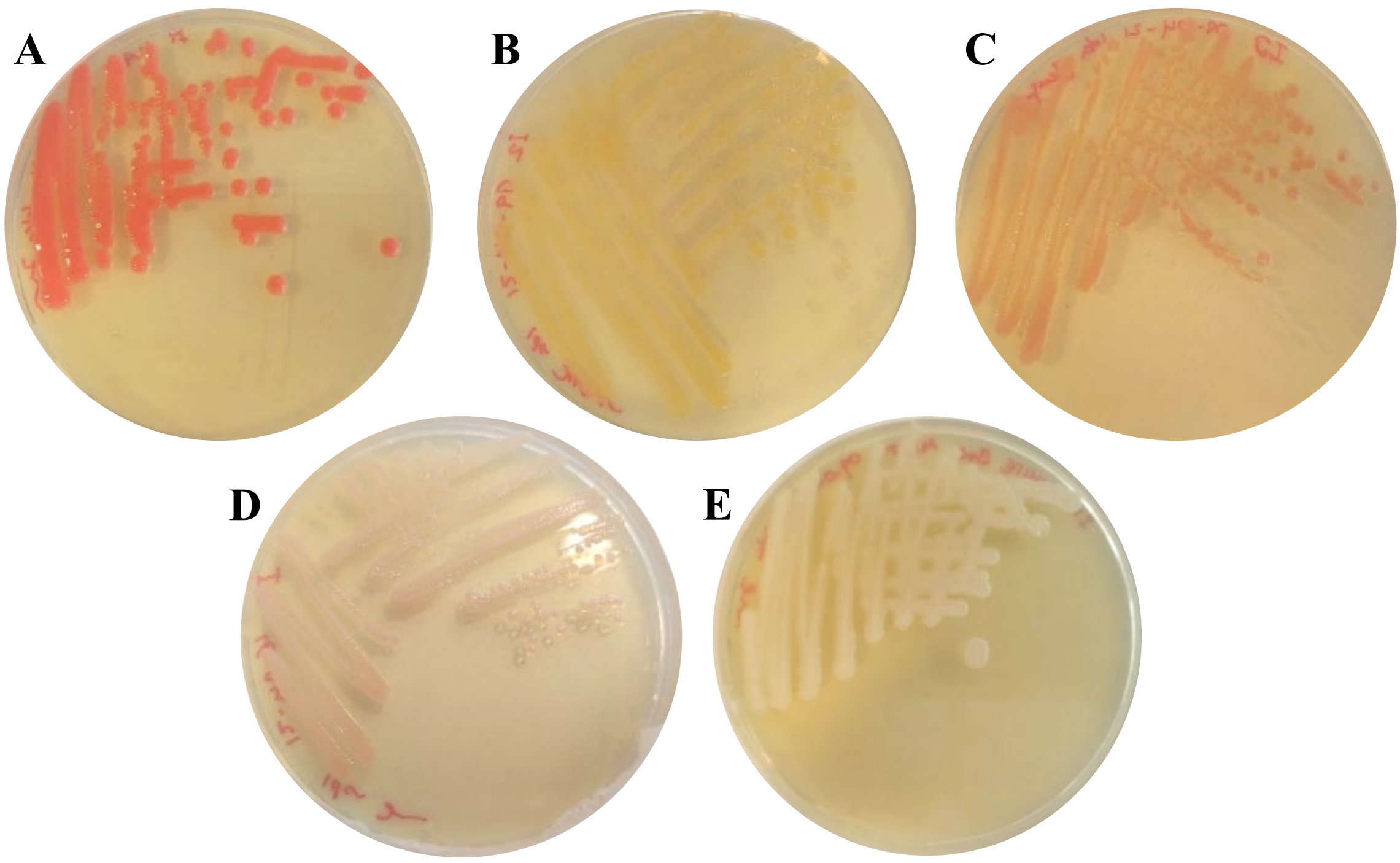

Figure S7. The five isolates from the washed ant extract and transfer test. "A", "D", and "E" denotes the yeast (I1a), and the two bacteria I4 and I1b derived from the washed ant extract, respectively. "B" and "C" denote the bacteria I2 and I3 derived from the transfer test, respectively.

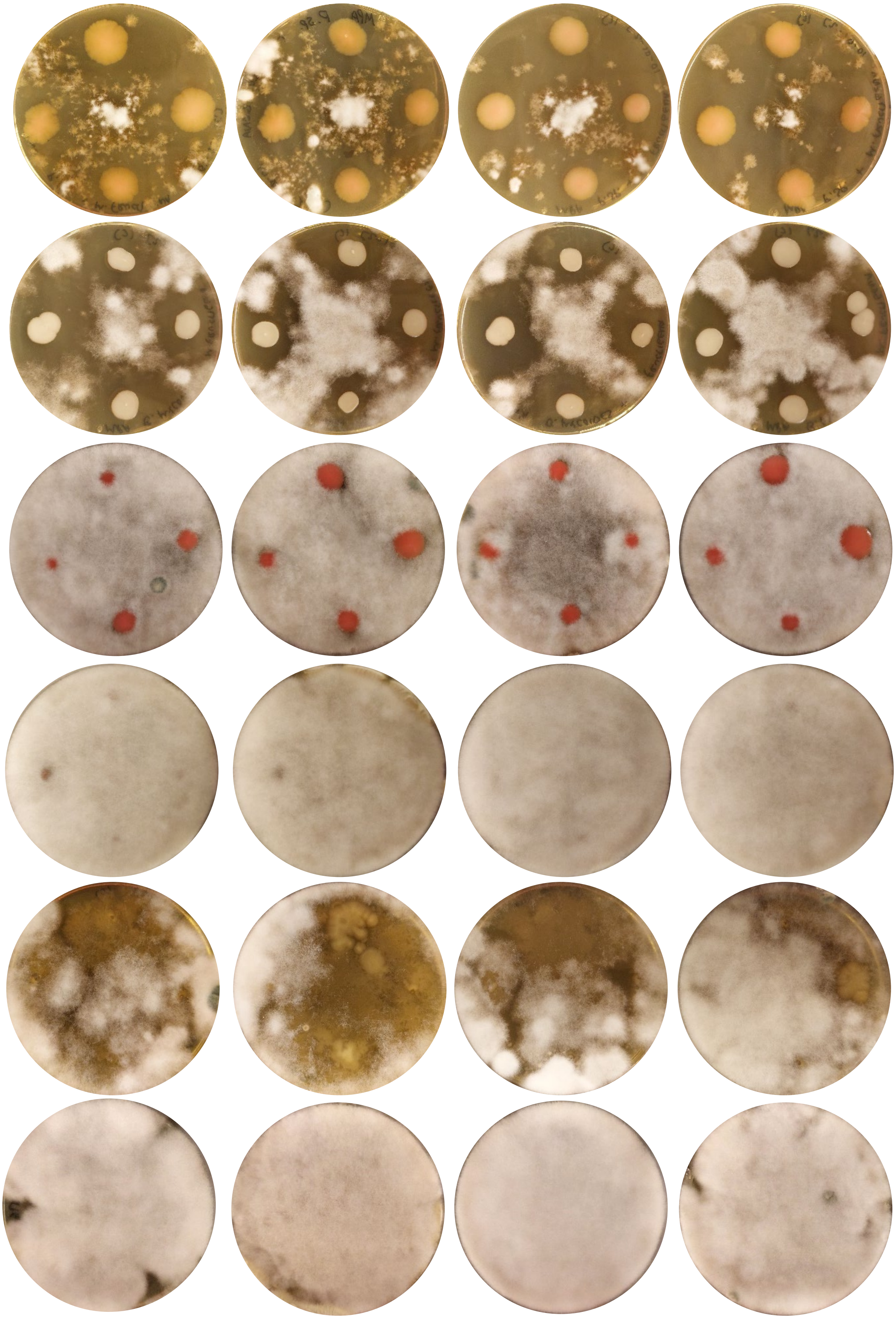

Figure S8. Inhibitory tests of the ant associated microorganisms against *M. fructigena*. First to fifth row show isolate I3 (*Pseudomonas* sp.), I1b (*Bacillus mycoides*), I1a, I4, and I2. The sixth row is the control plates containing only *M. fructigena*.

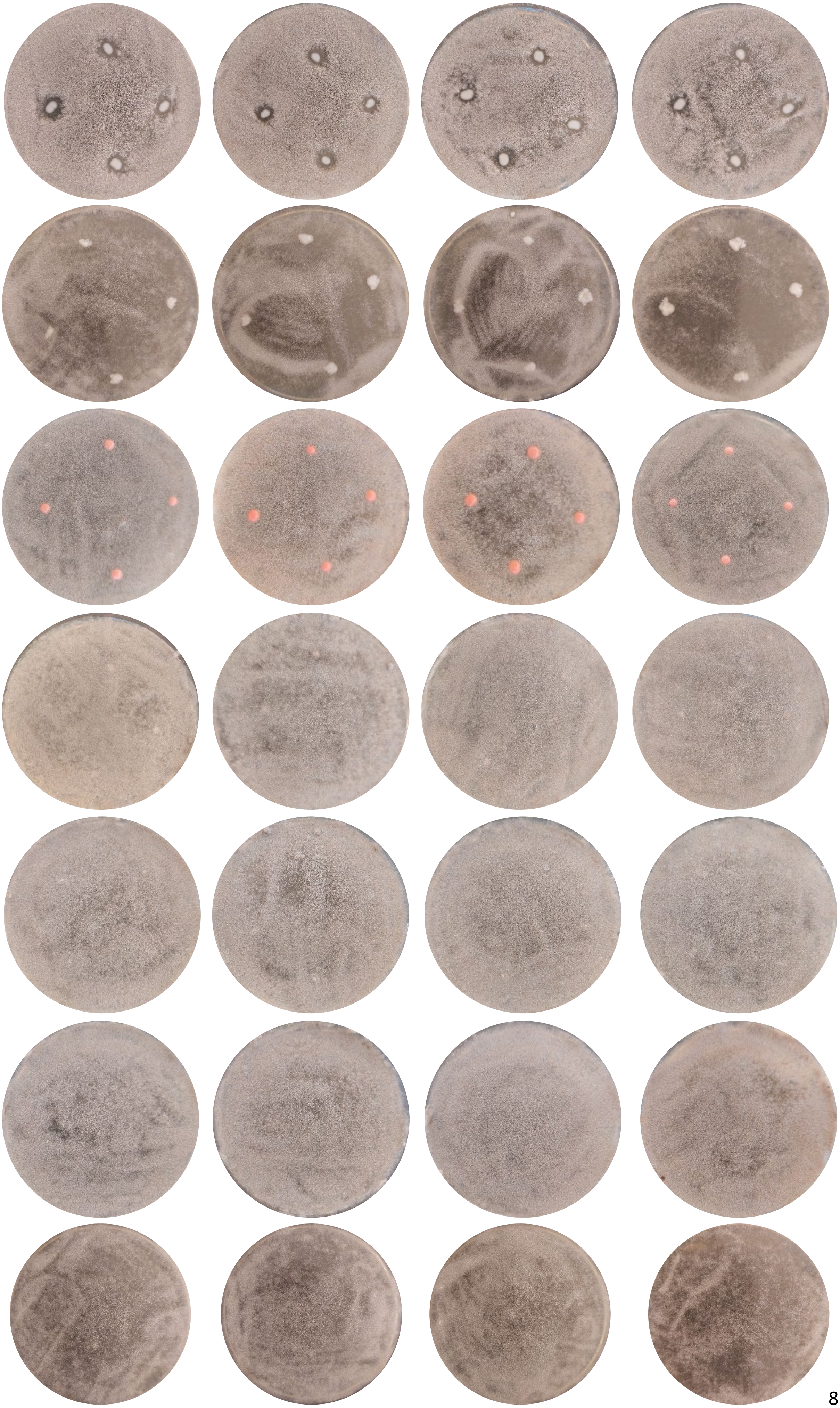

Figure S9. Inhibitory tests of the ant associated microorganisms against *Botrytis cinerea*. First to fifth row show isolate I3 (*Pseudomonas* sp.), I1b (*Bacillus mycoides*) grown on MPA, I1a, I4, and I2, respectively. The sixth row is the control plates containing only *B. cinerea* on PDA, while the seventh row is control plates grown on MPA.

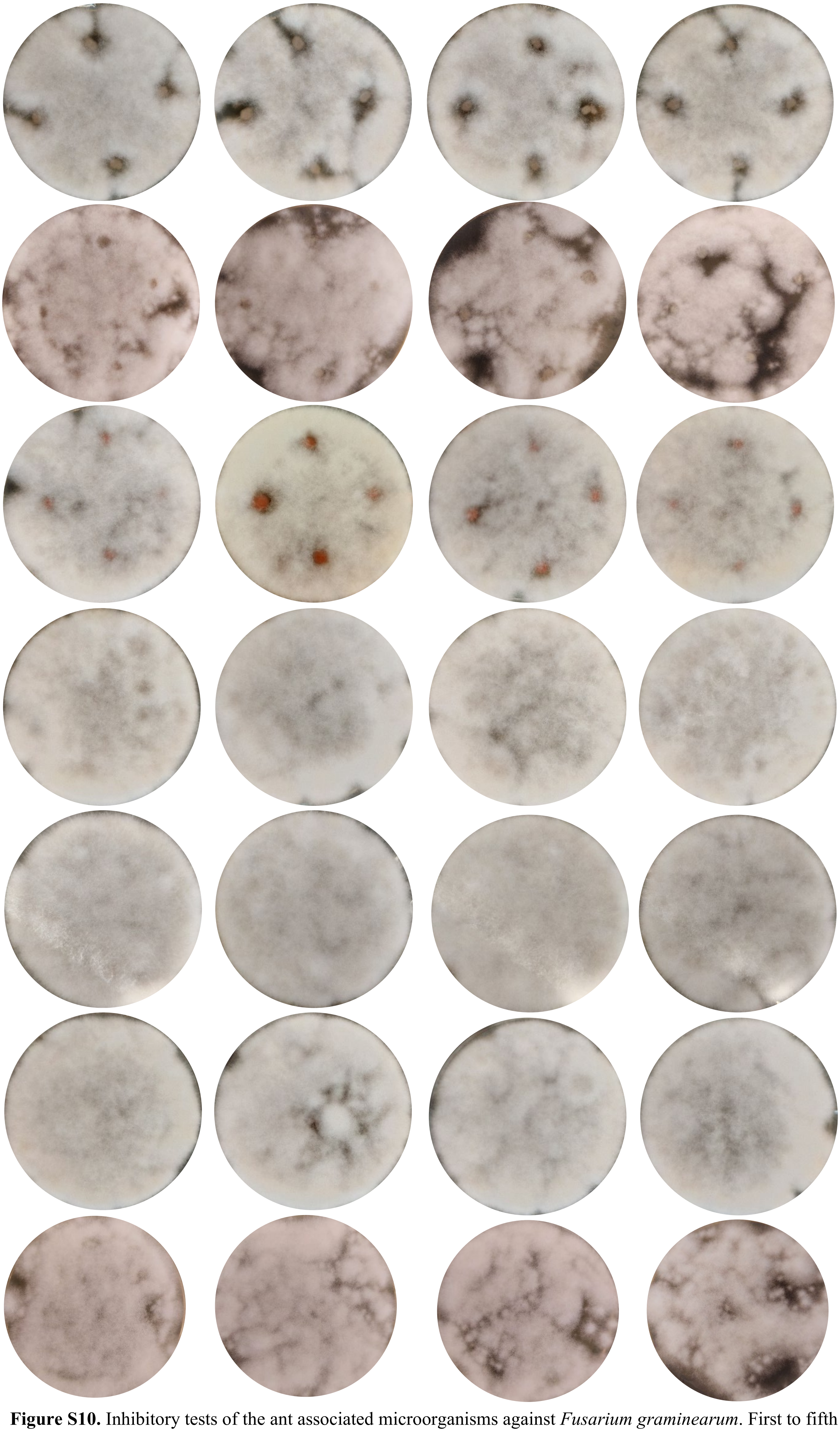

Figure S10. Inhibitory tests of the ant associated microorganisms against *Fusarium graminearum*. First to fifth row show isolate I3 (*Pseudomonas* sp.), I1b (*Bacillus mycoides*) on MPA, I1a, I4, and I2, respectively. The sixth row is the control plates containing only *F. graminearum* on PDA, while the seventh row is control plates grown on MPA.

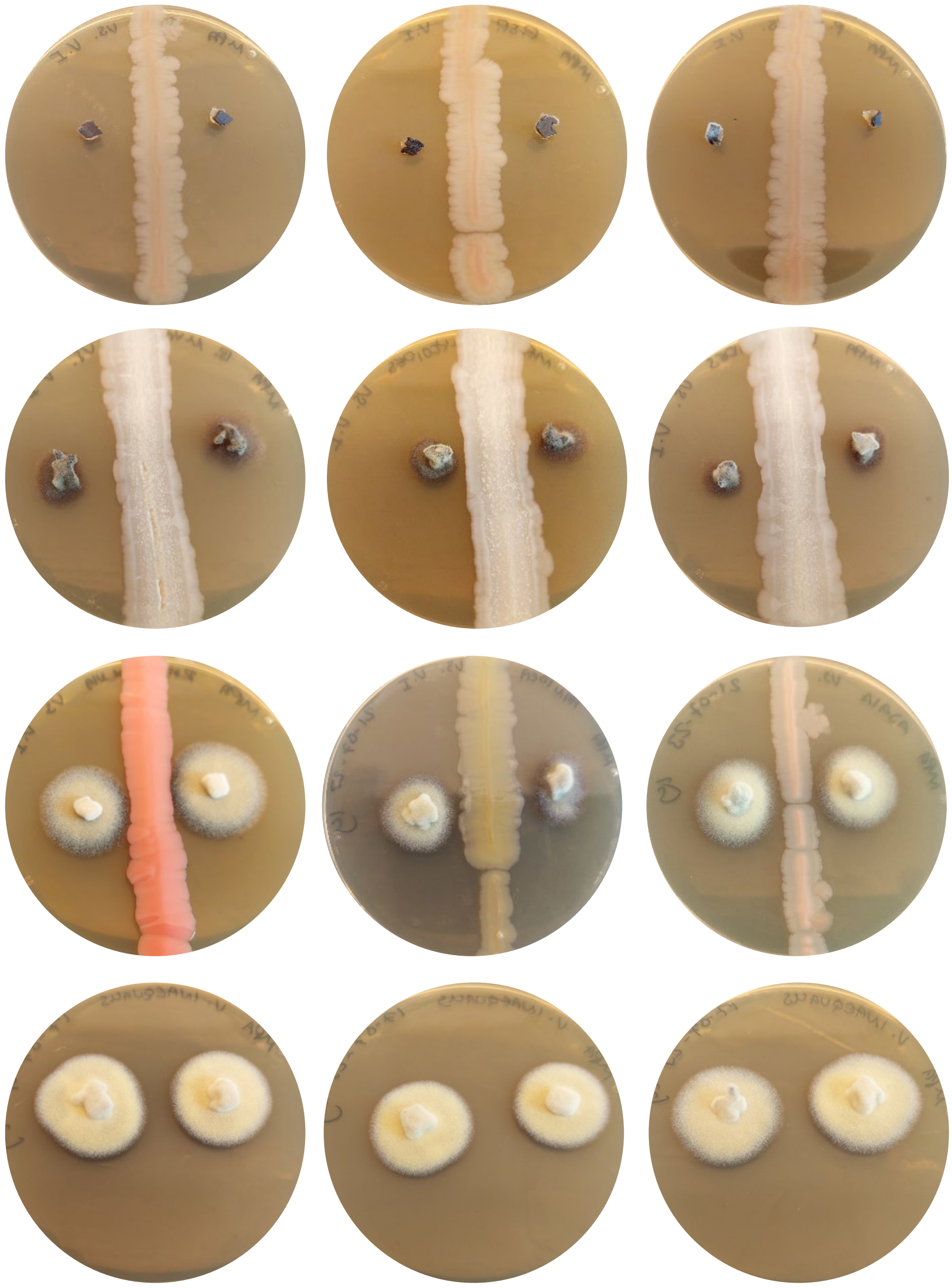

Figure S11. Inhibitory tests of the ant associated microorganisms against *Venturia inaequalis*. First to second row show isolate I3 (*Pseudomonas* sp.) and I1b (*Bacillus mycoides*), respectively, while the third row contains isolate I1a, I4, and I2, from left to right. The fourth row is the control plates containing only *V. inaequalis*.

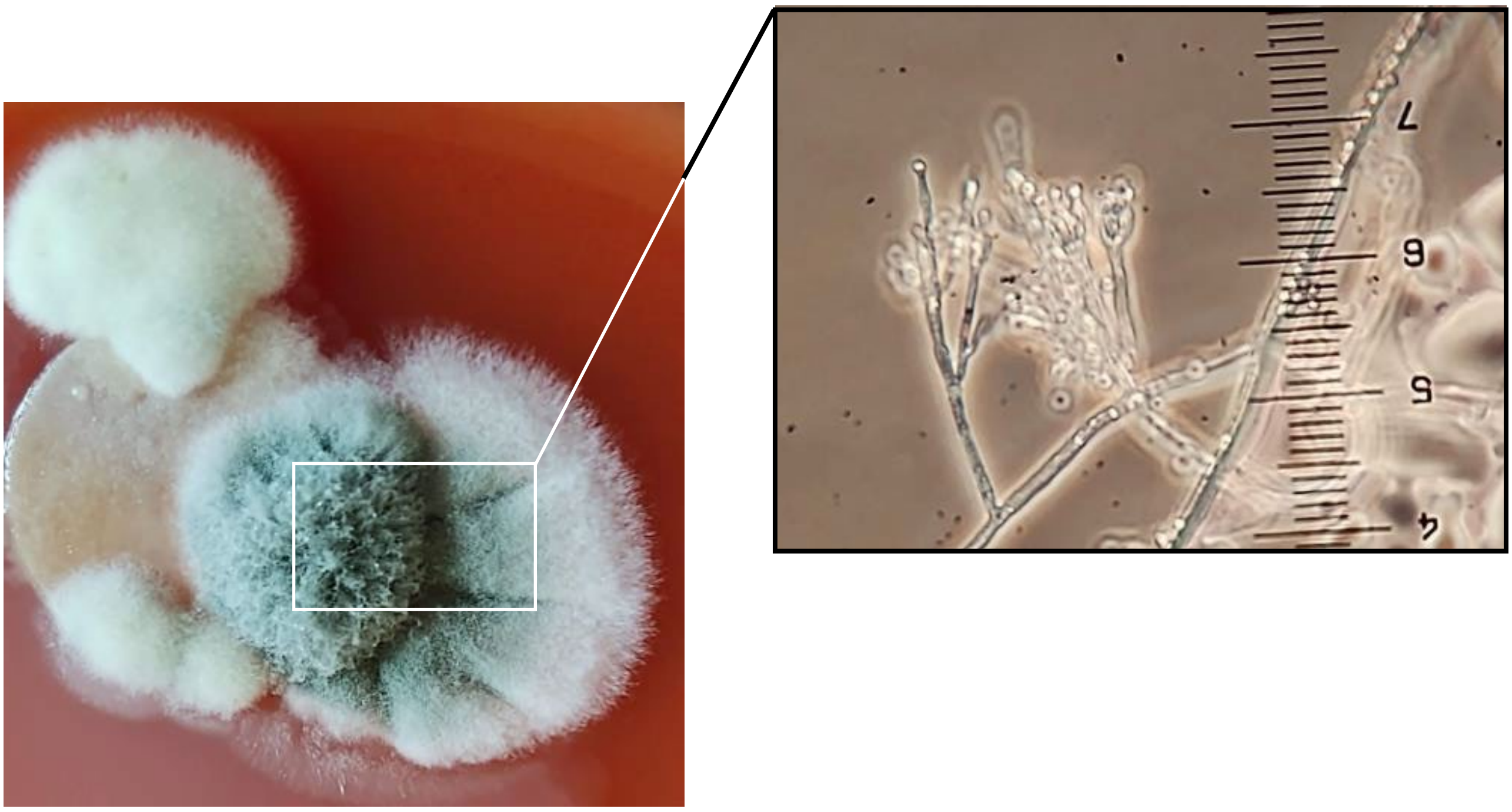

Figure S12. Close-up of the fungus believed to be *Penicillium* sp., found in the crushed ant extract. Right-side of the figure shows the conidiophores of the fungus enhanced using a microscope. The brush-like structure of the conidiophores is very similar to that of other *Penicillium* species.

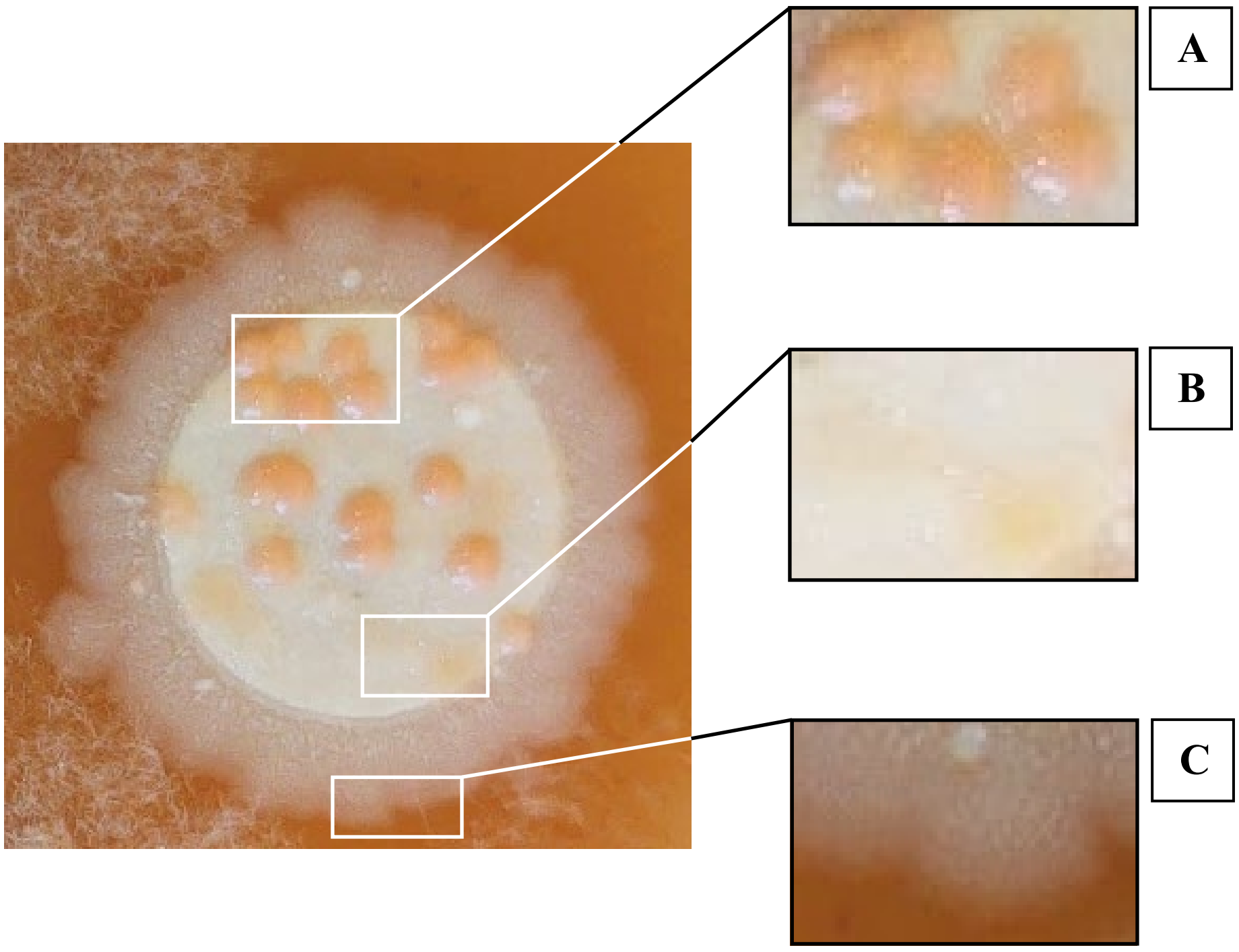

Figure S13. Bacteria and yeast dominant in washed ant extracts. "A" shows the yeast, I1a (most closely related to *Rhodotorula alborubescens*), "B" shows isolate I4 (most closely related to *Asaia spathodeae*), "C" shows isolate I1b (most closely related to (*Bacillus mycoides*)

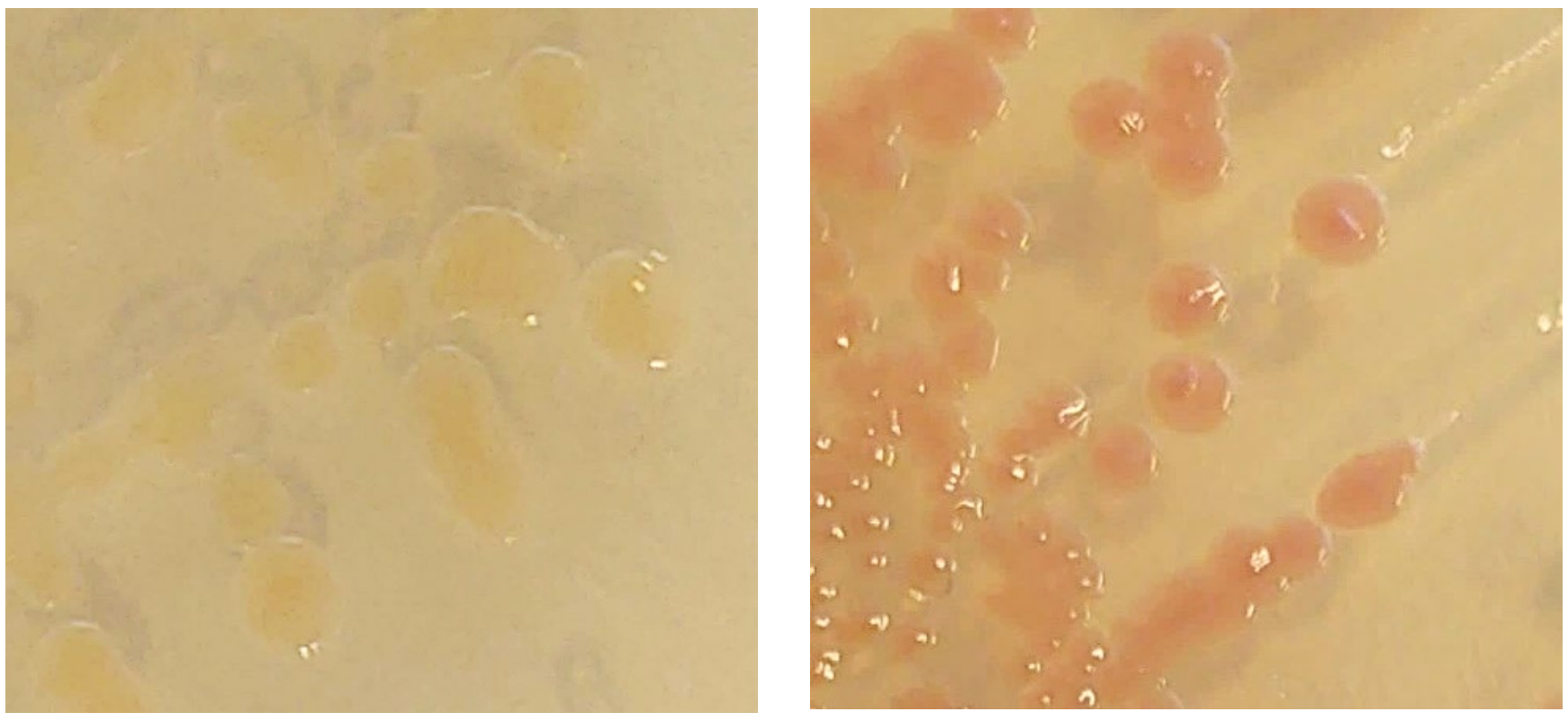

Figure S14. Bacteria found in the transfer test. The left-side picture shows isolate I2 (most closely related to *Scandinavium goeteborgense/Pantoea endophytica*) while the right-side picture shows isolate I3 (*Pseudomonas* sp.)

R Script for statistical analyses

```
#24 hour live ant activity
library(RcmdrMisc)
library(brms)

mydata<-readXL("24hour.xlsx",rownames=FALSE,header=TRUE) #loading the data
mydata$Treatment=as.factor(mydata$Treatment) #defining the variables
mydata$Inhibition=as.factor(mydata$Inhibition)


fit1=brm(Inhibition~Treatment, data = mydata, family = bernoulli(link = "logit"), prior =
c(set_prior("normal(0,100)",class = "b")), chains = 4,iter = 20000)
summary(fit1)
plot(fit1)


#Crushed ant extract
library(RcmdrMisc)
library(brms)

mydata2<-readXL("Crushedant.xlsx",rownames=FALSE,header=TRUE)
mydata2$Treatment=as.factor(mydata2$Treatment)
mydata2$Inhibition=as.factor(mydata2$Inhibition)

fit2=brm(Inhibition~Treatment, data = mydata2, family = bernoulli(link = "logit"), prior =
c(set_prior("normal(0,100)",class = "b")), chains = 4,iter = 20000)
summary(fit2)
plot(fit2)
```

#Washed ant extract

```
library(RcmdrMisc)
library(brms)

mydata3<-readXL("Washedant.xlsx",rownames=FALSE,header=TRUE)
mydata3$Treatment=as.factor(mydata3$Treatment)
mydata3$Inhibition=as.factor(mydata3$Inhibition)

fit3=brm(Inhibition~Treatment, data = mydata3, family = bernoulli(link = "logit"), prior =
c(set_prior("normal(0,100)",class = "b")), chains = 4,iter = 20000)
summary(fit3)
plot(fit3)
```
